# 3T MR imaging: diffusion-weighted and dynamic contrast-enhanced - relationship of apparent diffusion coefficient value and maximum percentage enhancement in invasive lobular carcinoma of the breast

**DOI:** 10.1186/bcr3277

**Published:** 2012-11-09

**Authors:** ID Lyburn, HC Russell, J Searle, C Croucher, DO Hall

**Affiliations:** 1 Thirlestaine Breast Centre, Cheltenham, UK; 2 Cheltenham Imaging Centre, Cheltenham, UK

## Introduction

In breast MR imaging, diffusion-weighted imaging (DWI) and dynamic contrast-enhanced (DCE) studies are being increasingly utilised in assessing malignancy. We investigated potential relationships between these two techniques in cases of proven invasive lobular carcinoma (ILC).

## Methods

Thirty-two consecutive cases of ILC underwent 3T breast MR imaging as part of routine staging - retrospective analysis was undertaken. Diffusion-weighted imaging using three b values (0, 500 and 1,000 seconds/mm^2^) was performed prior to dynamic contrast-enhanced imaging in each case. Post processing included obtaining maximum intensity projections and multiplanar reconstruction. Regions of interest (ROI) were placed over the index (presenting) lesion. In cases of multifocality/multicentricity, the most conspicuous enhancing lesion was interrogated. Enhancement kinetics were evaluated from the dynamic contrast-enhanced images: the percentage of signal intensity increase within the first 2 minutes after administrating contrast agent relative to pre-contrast signal intensity was calculated and the morphology of the curve for 6 minutes after contrast administration was analysed. The same size of ROI was placed over the lesion site on the DWI and the apparent diffusion coefficient (ADC) map was calculated.

## Results

Age range, 34 to 74 years; index lesion size, 4 to 78 mm. For type 2 and type 3 curves, signal increase was 128 to 306%; ADC mean 1.29 × 10^-3 ^mm^2^/second. In type 1 curves, signal increase was 82 to 93%; ADC mean 0.85 × 10^-3 ^mm^2^/second. In four cases (with type 1 or type 2 curves) no lesions with restricted diffusion were apparent.

## Conclusion

The percentage signal intensity increase on DCE is higher in cases with measurable ADC values than without. ILC with type 1 or type 2 curves may not be visible on DWI.

